# Neuroprotective Effect of HIF Prolyl Hydroxylase Inhibition in an In Vitro Hypoxia Model

**DOI:** 10.3390/antiox9080662

**Published:** 2020-07-24

**Authors:** Maria Savyuk, Mikhail Krivonosov, Tatiana Mishchenko, Irina Gazaryan, Mikhail Ivanchenko, Anna Khristichenko, Andrey Poloznikov, Dmitry Hushpulian, Sergey Nikulin, Evgeny Tonevitsky, Guzal Abuzarova, Elena Mitroshina, Maria Vedunova

**Affiliations:** 1 Department of Neurotechnology, Institute of Biology and Biomedicine, Lobachevsky State University of Nizhny Novgorod, 23 Gagarin Ave., Nizhny Novgorod 603950, Russia; mary.savyuk@bk.ru (M.S.); saharnova87@mail.ru (T.M.); helenmitroshina@gmail.com (E.M.); 2 Department of Applied Mathematics, Lobachevsky State University of Nizhny Novgorod, 23 Gagarin Ave., Nizhny Novgorod 603950, Russia; mike_live@mail.ru (M.K.); ivanchenko.mv@gmail.com (M.I.); 3 P. A. Hertsen Moscow Oncology Research Center, Branch of the National Medical Research Radiological Center, Ministry of Health of the Russian Federation, Moscow 125284, Russia; igazaryan@gmail.com (I.G.); hristik14@gmail.com (A.K.); or apoloznikov@hse.ru (A.P.); hushpulian@gmail.com (D.H.); abuzarova_mnioi@bk.ru (G.A.); 4 Chemical Enzymology Department, Chemistry Faculty, M. V. Lomonosov Moscow State University, Moscow 119992, Russia; 5 Faculty of Biology and Biotechnologies, Higher School of Economics, Moscow 101000, Russia; snikulin@hse.ru; 6 School of Biomedicine, Far Eastern Federal University, Vladivostok 690091, Russia; 7 Development Fund of the Innovation Science and Technology Center “Mendeleev Valley”, Moscow 125480, Russia; etonev@muctr.ru

**Keywords:** hypoxia-inducible factor (HIF), HIF prolyl hydroxylase, prolyl hydroxylase (PHD), neuradapt, primary hippocampal cultures, functional neural network activity

## Abstract

A novel potent analog of the branched tail oxyquinoline group of hypoxia-inducible factor (HIF) prolyl hydroxylase inhibitors, neuradapt, has been studied in two treatment regimes in an in vitro hypoxia model on murine primary hippocampal cultures. Neuradapt activates the expression of HIF1 and HIF2 target genes and shows no toxicity up to 20 μM, which is more than an order of magnitude higher than its biologically active concentration. Cell viability, functional activity, and network connectivity between the elements of neuronal networks have been studied using a pairwise correlation analysis of the intracellular calcium fluctuations in the individual cells. An immediate treatment with 1 μM and 15 μM neuradapt right at the onset of hypoxia not only protects from the death, but also maintains the spontaneous calcium activity in nervous cells at the level of the intact cultures. A similar neuroprotective effect in the post-treatment scenario is observed for 15 μM, but not for 1 μM neuradapt. Network connectivity is better preserved with immediate treatment using 1 μM neuradapt than with 15 μM, which is still beneficial. Post-treatment with neuradapt did not restore the network connectivity despite the observation that neuradapt significantly increased cell viability at 1 μM and functional activity at 15 μM. The preservation of cell viability and functional activity makes neuradapt promising for further studies in a post-treatment scenario, since it can be combined with other drugs and treatments restoring the network connectivity of functionally competent cells.

## 1. Introduction

Hypoxia is a widespread pathological process accompanying various diseases including ischemia, trauma, diabetes mellitus, Alzheimer’s disease, etc. Even a short-term hypoxia can lead to functional and structural changes in tissues and organs. The most sensitive organ to oxygen deprivation is brain. Alterations in oxygen availability affect synaptic transmission in neurons and disrupt neural networks [1]. Studies on protective mechanisms compensating for oxygen deficiency focus on the oxygen sensor system controlled by a family of hypoxia-inducible factors (HIFs) [2]. HIFs are heterodimers consisting of constitutively expressed β-subunit, insensitive to oxygen, and hypoxia-regulated α-subunit [3]. The stability of the latter is regulated by hydroxylation with oxygen via a reaction catalyzed by HIF prolyl hydroxylases (HIF PHD), a group of non-heme iron α−ketoglutarate (αKG)-dependent dioxygenases [4]. HIF-1 is a major transcription factor in oxygen homeostasis [5].

In normoxia, hydroxylation of Pro 564 makes HIF-1α subunit recognizable for ubiquitin ligase complex, which labels HIF-1α for proteasomal degradation. In hypoxia, HIF-1α subunit is stabilized, associates with β-subunit, and the dimeric HIF protein translocates to the nucleus, where it binds to a hypoxia-responsive element in hundreds of genes executing an anti-hypoxic genetic program. HIF target genes include proteins involved in angiogenesis, erythropoesis, glycolysis, iron and glucose transport, proliferation, cell survival, and vascular remodeling [6,7]. Pharmacological inhibition of PHD activity is supposedly neuroprotective and becomes a popular trend in strategies for ischemic injury correction [8,9,10,11]. However, the available literature is largely supportive of PHD inhibitors benefits in pre-treatment rather than post-treatment for various models of ischemia. A promising exception to this rule is adaptaquin, which demonstrated post-treatment neuroprotection in in vivo models of hemorrhagic stroke [12].

In contrast to other tissues and organs, brain functions, from simple reflex responses to complex cognitive processes, such as memory and thinking, are determined by the neural network activity. Even a relatively simple neural network has a large number of invariant solutions, assigned tasks, multiple responses and adaptive reactions. A neural network orchestrates the whole body adaptation to the dynamically changing environment by regulating the amplitude of synaptic transmission and reorganizing functional connections. Investigational neuroprotective compounds have to undergo in vitro testing with respect to their impact not only on neuronal viability, but on the neural network structure and activity. Functional calcium imaging is one of the techniques used to study the network activity. However, standard approaches to analyze the data on functional calcium activity do not allow complex relationships between elements of neuron-glial network to be evaluated. The network data analysis based on correlation of time-series and spatio-temporal pattern recognition can solve this problem at the cellular and subcellular levels and answer fundamental questions concerning the peculiarities of adaptive functional reorganization of neural networks in response to stress.

The current study aims at testing a novel PHD inhibitor [13], an improved analog of adaptaquin [14], with respect to its neuroprotective properties in an in vitro hypoxia model, with emphasis on the functional neural network activity.

## 2. Materials and Methods

### 2.1. Reporter Assay

**Figure 1:**
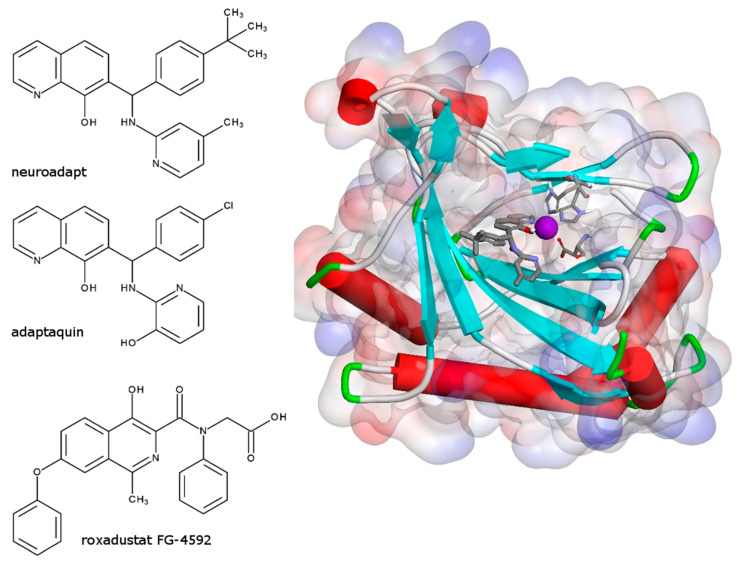
Chemical structures of HIF PHD pan-inhibitors, e.g., neuradapt, adaptaquin and roxadustat; and neuradapt docking into the PHD2 crystal structure (2G19.pdb). The active site iron shown in deep purple is coordinated by two histidine and one asparatate residues; the neuradapt oxyquinoline ring provides two ligands to the iron. Docking performed with Discovery Studio 3.0.

Compound 4896-3212 (named neuradapt) was obtained from ChemDiv Research Institute (Khimki, Russia), adaptaquin and FG-4592 (roxadustat) were purchased from Cayman Chemical (Ann Arbor, MI, USA). Compound structures are shown in Figure 1. HIF1 ODD-luc/SH-SY5Y cell-based reporter assay was performed as described in [14]. Drug aliquots were prepared in DMSO, and added to the reporter cell line as 2 μL aliquots. Luciferase activity was measured 3 h post-incubation with a drug. Experiments were performed in triplicate. 

### 2.2. Real Time PCR

The SH-SY5Y cells were grown in a 6-well microplate till 500,000 cell/well density, and then the studied compounds (10 μM neuradapt or 30 μM roxadustat) were added, and the cells were incubated for 5 h. The cells were collected, washed, and lysed with QIAzol (Qiagen, Germany). Lysates were used to isolate total RNA using a MiRNEasy Micro Kit (Qiagen, Germany) in accord with the manufacturer’s instructions. The concentration of purified RNA was determined using a Nanodrop ND-1000 spectrophotometer (ThermoFisher Scientific, USA). Reverse transcription was performed using a MMLV RT Kit (Evrogen, Russia) in accord with the manufacturer’s protocol and using a random primer Random(dN)10 (Evrogen, Russia): 60 min incubation at 42 °C followed by 10 min incubation at 70 °C. A representative panel of HIF-target genes and 4 reference genes was used (Table S1).

Primers were selected using mRNA sequences from GenomeBrowser database using Primer3 [15] and Primer-BLAST software [16]. The primers used are shown in Supplementary Table S1. Real-time PCR was performed in a 96-well microplate using a qPCRmix-HS SYBR kit (Evrogen, Russia) and a DNA amplificator (DNA-Technology, Russia). Amplification included 10 min denaturation at 94 °C, 40 cycles 20 s melting at 94 °C followed by 10 s annealing and 15 s elongation at 72 °C. Experiments were performed in triplicate. Ct values were obtained at 5000 fluorescence thresholds. Statistical significance of the differences in the expression was evaluated with REST 2009 software (v.2.0.13) [17].

Relative expression was calculated in accord with ΔΔCt method using the following formula, where ACTB, GAPDH, RPLP0 and POL2RF are reference genes, e.g., beta-actin, glyceraldehyde 3-phosphate dehydrogenase, 60S acidic ribosomal protein P0, and polymerase (RNA) II (DNA directed) polypeptide F, respectively:

(1) $$R=2^{\left[{\Delta \mathrm{Ct}}_{\mathrm{target}}-\frac{1}{4}\times \left({\Delta \mathrm{Ct}}_{\mathrm{ACTB}}+{\Delta \mathrm{Ct}}_{\mathrm{GAPDH}}+{\Delta \mathrm{Ct}}_{\mathrm{RPLP}0}+{\Delta \mathrm{Ct}}_{\mathrm{POL}2\mathrm{RF}}\right)\right]}$$

(2) $${\Delta \mathrm{Ct}}_{\mathrm{target}}={\mathrm{Ct}}_{\mathrm{target},\mathrm{control}}-{\mathrm{Ct}}_{\mathrm{target},\mathrm{sample}}$$

(3) $${\Delta \mathrm{Ct}}_{\mathrm{ACTB}}={\mathrm{Ct}}_{\mathrm{ACTB},\mathrm{control}}-{\mathrm{Ct}}_{\mathrm{ACTB},\mathrm{sample}}$$

(4) $${\Delta \mathrm{Ct}}_{\mathrm{GAPDH}}={\mathrm{Ct}}_{\mathrm{RPLP}0,\mathrm{control}}-{\mathrm{Ct}}_{\mathrm{GAPDH},\mathrm{sample}}$$

(5) $${\Delta \mathrm{Ct}}_{\mathrm{RPLP}0}={\mathrm{Ct}}_{\mathrm{RPLP}0,\mathrm{control}}-{\mathrm{Ct}}_{\mathrm{RPLP}0,\mathrm{sample}}$$

(6) $${\Delta \mathrm{Ct}}_{\mathrm{POL}2\mathrm{RF}}={\mathrm{Ct}}_{\mathrm{POL}2\mathrm{RF},\mathrm{control}}-{\mathrm{Ct}}_{\mathrm{POL}2\mathrm{RF},\mathrm{sample}}$$

### 2.3. Ethics Statement

All experimental procedures have been approved by the Bioethics Committee of Lobachevsky University and carried out in accord with Act 708n (23 082010) of the Russian Federation National Ministry of Public Health, which states the rules of laboratory practice for the care and use of laboratory animals, and Council Directive 2010/63 EU of the European Parliament (22 September 2010) on the protection of animals used for scientific purposes. Pregnant C57BL/6 mice (day of gestation 18) were sacrificed by cervical vertebra dislocation.

### 2.4. Isolation of Murine Primary Hippocampal Cultures

Hippocampal cells were obtained from mice embryos and cultured on coverslips (18 × 18 mm) pretreated with polyethyleneimine solution (1 mg/mL) (Sigma-Aldrich, P3143, Steinheim Germany ) according to the previously developed protocol described in [18]. Isolation of embryonic hippocampi was performed in Ca^2+^- and Mg^2+^-free phosphate-buffered saline (PBS) with subsequent enzymatic digestion with 0.25% trypsin-ethylenediaminetetraacetic acid (EDTA, Invitrogen, 25200-056) for 20 min. After centrifugation (800 rpm for 3 min), the pellet of dissociated cells was seeded on coverslips at approximate initial density of 7000–9000 cells/mm^2^. The primary hippocampal cultures were grown in Neurobasal medium (Invitrogen, 21103-049, CA, USA) supplemented with 2% B27 (Invitrogen, 17504-044), 0.5 mM L-glutamine (Invitrogen, 25030-024, CA, USA), and 0.4% fetal bovine serum (FBS; PanEco, K055, Russia) under constant conditions of 35.5 °C, 5% CO^2^ and a humidified atmosphere in a Binder C150 incubator (BINDER GmbH, Tuttlingen, Germany). A half-replenishment of the medium was performed once in every three days.

### 2.5. Acute Normobaric Hypoxia Model

Acute normobaric hypoxia was modeled on day 14 of cultures development in vitro (DIV) by replacing a normoxic culture medium with a medium containing low oxygen for 10 min. The oxygen was displaced from the medium in a sealed chamber with an inert gas (argon). A partial pressure of oxygen in water vapor saturated air at 37 °C is 147 mmHg and corresponds to 0.207 mM O_2_ in pure water. It becomes slightly lower in physiological solutions (for example, 0.190 mM O2 in mitochondria assay buffers). However, a CO_2_-incubator gas mixture contains 5% CO_2_ in addition to H_2_O vapor. CO_2_ may decrease the content of dissolved oxygen. Therefore, we determined the actual concentration of dissolved oxygen in the cultural medium under the experimental conditions. The dissolved oxygen in the culture medium was assayed using Winkler’s method [19]. Briefly, oxygen concentration in the medium is assayed by iodometric titration. The method is based on quantitative oxidation of Mn(II) hydroxide to Mn(IV) hydroxide with dissolved oxygen. Upon acidification, Mn(IV) hydroxide is quantitatively reduced by iodide yielding iodine. The latter is titrated with sodium thiosulfate using starch as an indicator.

Concentration of dissolved oxygen in the normoxic cultural medium was found equal to 3.26 mL/L, which at 35.5 °C corresponded to 0.140 mM O_2_, or ca. 100 mmHg. An increase in pCO_2_ creates the conditions close to physiological ones. The oxygen concentration determined in the cell culture almost exactly corresponds to the oxygen content in the arterial blood, which is in the range 3.1–3.26 ml/L, or 100–108 mmHg [20]. In the cited paper, the authors also stated that a 2-fold drop in oxygen tension in femoral artery is sufficient to induce moderate hypoxia. In Johnston et al. [21], the level of 115 mmHg O_2_ is considered a moderate hyperoxia. Moreover, lower pO_2_ values are below K_m_(O_2_) for HIF prolyl hydroxylase enzyme and will cause partial activation of antihypoxic program in the absence of the drug. Under such conditions, it would be difficult to judge on the drug effect. Direct assay of pO_2_ in brain of TBI patients showed that hypoxia level of 10–15 mmHg was associated with increased mortality, whereas pO_2_ less than 6 mmHg was not compatible with life [22]. Based on above observations, we chose 100 mmHg pO_2_ as normoxia, and 11 mmHg pO_2_ (0.37 ml/L) as acute hypoxia aiming to be (a) within the range of physiologically relevant concentrations, but (b) with a sufficient change (10-fold) in oxygen concentration to evaluate the effect of HIF prolyl hydroxylase inhibitor. 

The oxygen concentration in the culture medium was decreased from 3.26 ml/L to 0.37 ml/L [23]. After 10 min incubation, the hypoxic medium was replaced by a complete culture medium.

### 2.6. Pharmacological Treatment

**Figure 2:**
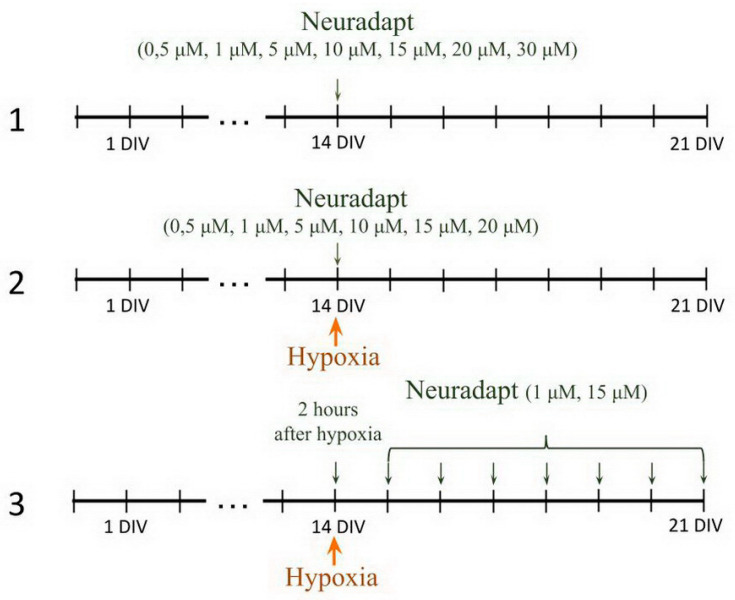
Experimental design flowchart. Step 1, Toxicity evaluation; Step 2, Immediate treatment regime with neuradapt (at the onset of hypoxia); Step 3, Post-treatment regime with neuradapt (2 h post hypoxia, and continues for 7 days).

Compound 4896-3212 (neuradapt) was used to inhibit HIF prolyl hydroxylase. The experimental scheme included three steps (Figure 2):

1. PHD inhibitor neuradapt cytotoxicity assessment. The following drug concentrations were analyzed: 0.5 µM, 1 µM, 2 µM, 5 µM, 10 µM, 15 µM, 20 µM, 30 µM. The solvent, dimethyl sulfoxide (DMSO) (Sigma-Aldrich, D8418, Steinheim, Germany), was used in the control group;
2. Immediate treatment. To study the effect of neuradapt on cell viability and functional parameters of neuron-glial networks in an in vitro hypoxia model, 0.5 µM, 1 µM, 2 µM, 5 µM, 10 µM, 15 µM, and 20 µM were added to the hypoxic culture medium and to the normoxic medium used for re-oxygenation;
3. Post-treatment. To evaluate the post-hypoxia neuroprotective action of neuradapt, 1 µM or 15 µM drug was applied to the culture medium daily starting 2 h post-hypoxia modeling and continued for the following 7 days.

### 2.7. Cell Viability Assay

The viability of primary hippocampal cultures was expressed as a ratio of the number of the dead cells nuclei stained with propidium iodide (Sigma-Aldrich, P4170, Steinheim Germany) to the total number of cells stained with bisBenzimide (Sigma-Aldrich, B1155, Steinheim, Germany). Propidium iodide and bisBenzimide at concentrations of 5 μg/mL and 1 μg/mL, respectively, were added to the culture 30 min before registration. The stained cultures were observed using a ZEISS Observer A1 inverted fluorescence microscope (Carl Zeiss, Oberkochen, Germany) with a 20×/0–2 Ph1 objective.

### 2.8. Calcium Imaging

To characterize the functional state of calcium homeostasis in the cells, functional calcium activity imaging was performed with a fluorescent calcium-sensitive dye Oregon Green 488 BAPTA-1 AM (OGB-1) (Invitrogen, O-6807, CA, USA) on an LSM 510 confocal laser scanning microscope (Carl Zeiss, Oberkochen, Germany). The calcium imaging technique allowed visualization of the functional architecture of neuron-glial networks to be achieved at the cellular level. The calcium sensor, 0.4 μM OGB-1, was dissolved in DMSO with 4% pluronic F-127 (Invitrogen, P-3000 MP, CA, USA), added to the examined cultures, and incubated for 30 min in a CO_2_-incubator. The OGB1 fluorescence excitation wavelength was set at 488 nm with an argon laser, and the emission was recorded using a 500–530 nm filter. To evaluate the dynamics of changes in the intracellular calcium concentration, a time-series of confocal images was recorded. The registration rate used was 2 frames per second. The spontaneous calcium activity of primary hippocampal cultures was recorded on day 7 post-hypoxia. The following parameters were analyzed: the duration of calcium oscillations (the time period from the beginning to the end of an oscillation, s), the frequency of calcium oscillations (an average number of oscillations per min), and the percentage of working cells (the cells number with at least one recorded oscillation divided by the total cell number, %) [24].

### 2.9. Network Characteristics of Primary Hippocampal Cultures

To analyze the network characteristics, we used the previously developed algorithm for detection of calcium activity in the cells [25]. A pairwise correlation analysis based on fluctuations in the intracellular calcium level in the individual cells was carried out. A functional network was presented as a non-oriented graph with nodes corresponding to the cells, and the edges designating a significant correlation between the levels of calcium activity in the cells exceeding the threshold value (ρ > 0.3). This threshold value for neuronal activity detection was chosen based on consideration of the correlation levels of the cells in a primary astrocyte monoculture depending on the distance between the cells. In the absence of neurons, for the spontaneous calcium activity in the astrocyte monocultures, the correlation level between the distant cells does not exceed the minimum value of 0.3, which allows it to be selected as the maximum level of correlation in a network without neuronal activity. The correlation values of spontaneous calcium activity above 0.3 indicate the significant activity of a neuronal network. 

The average level of correlation between the adjacent cells shows the strength of physical connections required for network communication. Low values of this parameter (0.1 ± 0.01) indicate the absence of physically related outgrowths or lack of their activity. The normal value of the parameter (0.21 ± 0.08) implies a potentially consolidated network response. An average number of functional connections per cell is an important characteristic of network activity. This parameter describes the average number of cells whose calcium activity significantly correlates with the individual cell in the network. The absence or low average numbers of connections characterize the lack of neuronal activity in a neuronal culture. This event indicates the cell death or disruption in cell functioning. The percentage of correlated connections out of the total number of possible connections quantifies the used resource of significant correlations in the network. A 0% value means the complete absence of significant correlations. A 100% value characterizes the highest level of significant correlations attained in the network. An increase in the above two parameters indicates the presence of a significantly correlated long-term response in the cell network, whereas their low values indicate its absence.

### 2.10. Statistical Analysis

Quantitative results are presented as a mean ± standard mean error (SEM) for normal distributions, or as a median value and second and third interquartile range. Statistical analyses were performed using ANOVA and the Mann–Whitney test implemented in R 3.5 programming language or Sigma Plot 11.0 software (Systat Software, Inc.). The Tukey post hoc test was used as a post hoc test following ANOVA. At least three independent biological replicates were used for all experiments. Differences between groups were considered significant if the corresponding *p* value was less than 0.05.

## 3. Results

### 3.1. HIF Activating Properties of Neuradapt

**Figure 3:**
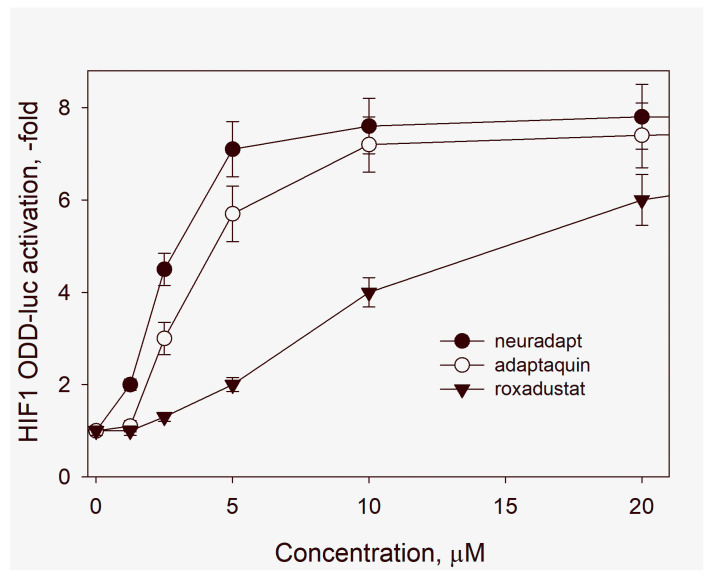
Cell-based reporter activation upon 3 h incubation with varied concentrations of HIF PHD inhibitors. Luminescence normalized to that in the absence of added inhibitors. Results presented as mean ± SD of triplicate measurement.

Peculiarities of the antihypoxic program triggered by a particular HIF PHD inhibitor depend on multiple factors stemming from the inhibitor specificity and chemical nature. Three isoforms of HIF PHD are known, however all available inhibitors can be classified as enzyme pan-inhibitors targeting all three enzyme isoforms. Three HIF isoforms are known, among those, highly homologous HIF-1 and HIF-2 isoforms are the best studied ones. All enzyme inhibitors, except adaptaquin [14], have been developed using an in vitro enzyme assay with recombinant HIF PHD2, and roxadustat (FG-4592) is no exception. Adaptaquin, as well as its optimized analog, neuradapt (compound 4896-3212) used in this study, have been developed using a cell-based reporter assay, HIF1 ODD-luc/SH-SY5Y cell line [14]. The comparison of adaptaquin, neuradapt, and roxadustat in the above reporter assay (Figure 3) demonstrates that the newly developed adaptaquin analog is 2–3 times more potent than adaptaquin itself, and an order of magnitude more potent than roxadustat. Hence, one may expect that neuradapt will be more effective in HIF stabilization and activation than roxadustat. 

To access the compounds potency and specificity with respect to HIF target genes, a representative PCR panel has been developed. HIF-1 and HIF-2 target genes largely overlap. However, each isoform exhibits some specificity for inducing a particular genetic program: HIF-1 triggers anaerobic glycolysis [26] and regulates the expression of genes responsible for intracellular pH maintenance and mitochondrial respiration [27], whereas HIF-2 target genes are linked to erythropoiesis, angiogenesis, cell cycle regulation, differentiation, and invasion [28]. The following genes specific for HIF-1 have been selected: phosphofructokinase (*PFKFB3*) [29,30], pyruvate kinase (*PKM*) [31,32], and Lon protease (*LONP1*) [33], the enzyme that catalyzes degradation of the subunit 4 of cytochrome c oxidase to adapt the latter activity to hypoxia. Genes specific for HIF-2 include erythropoietin (*EPO*) [34,35], cyclin D1 (*CCND1*) [36], matrix metalloproteinase 2 (*MMP2*) [32,37], and divalent metal transporter (*DMT1*) [38]. Common targets for both HIFs include vascular endothelial growth factor (*VEGFA*) and *RGS4* gene coding for a protein regulating signaling activity of G-proteins, which enhanced expression is specific for hypoxic neuroblastoma cells [39].

**Figure 4:**
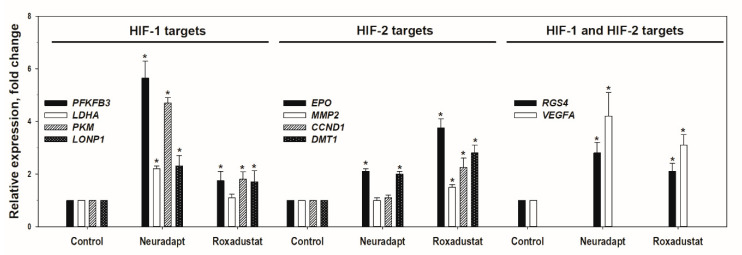
Relative expression of HIF target genes upon 5 h incubation of neuroblastoma SH-SY5Y cells with 10 μM neuradapt and 30 μM roxadustat, * *p* < 0.05.

As seen in Figure 4, neuradapt is more potent than roxadustat with respect to common genes, and definitely more specific for HIF1 target genes, whereas roxadustat is more specific for HIF2 targets such as *Epo* gene. The difference in action of these two inhibitors may originate from the differences in their chemical structure and mechanism of inhibition, namely, roxadustat mimics αKG binding mode in the active center and has only one “tail” attached to the isoquinoline ring. Roxadustat structure will not interfere with its binding to some other enzyme of this class, and in particular to HIF asparagine hydroxylase (the so called FIH, see 5OPC.pdb, FIH crystal structure with vadadustat, another HIF PHD inhibitor which has the mode of binding similar to roxadustat), which is responsible for fine tuning of HIF activity by hydroxylating HIF Asn 803 residue [40]. Neuradapt, in contrast to roxadustat, is exclusively specific for HIF PHD, since its “branched tail” in the 7th position of the oxyquinoline ring make its binding to the other enzymes of this family, and FIH in particular, impossible [14]. Therefore, testing neuradapt, especially at low micromolar concentrations, in in vitro hypoxia models will demonstrate the effects HIF PHD inhibition only on neuron–glial networks. 

### 3.2. Evaluation of Neuradapt Cytotoxicity

First, the range of non-toxic neuradapt concentrations has been determined by accessing the viability of primary hippocampal cultures in the normal state. A single application of the inhibitor was performed on day 14 in vitro grown cultures (DIV14). The formation of mature chemical synapses and stable neuronal network activity by DIV14 has been shown previously [41,42,43]. Hippocampus has a relatively simple cellular composition and is genetically predisposed to form local neural networks; therefore, the use of primary cultures obtained from this brain region is preferred for in vitro studies on neuron-glial networks. In addition, by day 14, the viable cells number differs from that at the early step of cultivation, as the formation of a complex neuron–glial network ensures the appearance of systemic reactions in response to various stress-factors, including toxic factors. Moreover, the neurons not engaged into neuron-glial networks and not forming sufficient number of connections die by day 14. As a result, the cell viability in primary hippocampal cultures on day 14 in the normal state equals to 93.64% ± 1.23%. Cytotoxicity assessment of neuradapt was performed on day 7 post-treatment, accounting for the known stepwise response of native neurons to toxins. The first step, an immediate response to a toxin, develops within 3 days; the second step targets the “borderline” cells which lost connections and thus, were excluded from the neural network and subject to apoptosis. As shown in Table 1, neuradapt in the 0.5–20 µM concentration range did not change the viability of primary hippocampal cultures compared to the intact group (Table 1). However, neuradapt at 30 μM concentration significantly (*p* ˂ 0.05, ANOVA) decreased (>10%) the number of viable cells. Therefore, neuradapt in the 1–20 μM concentration range showed no toxicity and could be used in in vitro model studies.

### 3.3. Evaluation of Neuradapt Neuroprotective Effects upon Immediate Administration

The neuroprotective potential of neuradapt was evaluated in a wide range of its non-toxic concentrations in an acute in vitro normobaric hypoxia model in primary hippocampal cultures. A significant decrease in the number of viable cells, down to 76.61% ± 2.67% by day 7 post-hypoxia, was observed, whereas the “Intact” group preserved 91.24% ± 0.75% viable cells (Table 2). 

The PHD inhibitor applied within the 0.5–20 μM concentration range significantly protects the cells from the hypoxia-induced damage bringing the survival level to the one for the intact group within the experimental error (Table 2). Thus, the novel PHD inhibitor exerts a pronounced neuroprotective effect in the in vitro model of acute normobaric hypoxia.

### 3.4. Neuradapt Effects in Post-Treatment Regime 

At the next step, we evaluated neuradapt post-treatment effects, namely, when the neuradapt addition started 2 h post-hypoxia and continued for consequent seven days. All existing methods for ischemic injury correction are limited to a “therapeutic window” period. In the best real life scenario, the anti-ischemic treatment may start 2 h post-episode of cerebral blood circulation disruption, but usually it begins much later. The secondary cell death beyond the ischemic lesion area develops within six days, giving an opportunity to inhibit apoptosis at later stages of post-ischemic complications. Two neuradapt concentrations, flanking the studied range of its non-toxic concentrations, 1 μM and 15 μM, were selected for post-treatment scenario.

As seen in Table 3, cell viability in the primary hippocampal cultures on day 7 post-hypoxia with daily applications of neuradapt was significantly higher than in the “hypoxia” group (73.11% ± 1.76%): 80.85% ± 1.41% for 1 μM and 80.70% ± 1.19% for 15 μM neuradapt, respectively (Table 3).

Despite the fact that neuradapt did not restore the cell viability to the “intact” group level, its post-hypoxia application did exhibit a protective effect compared to the non-treated “hypoxia” group. Thus, the significantly increased viability of primary hippocampal cells in a post-treatment regime with neuradapt truly indicates its neuroprotective effect and makes it a promising candidate for the development of new therapeutic agents working post-hypoxia and possibly post-ischemia.

### 3.5. Features of Functional Network Activity in Primary Hippocampal Cultures in the Post-Hypoxic Period

**Figure 5:**
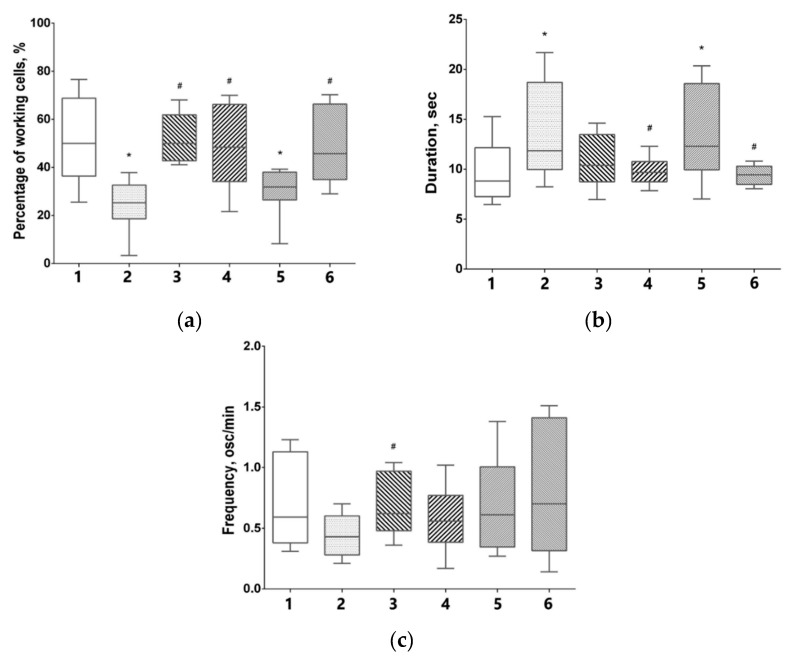
Key parameters of spontaneous Ca^2+^ activity in primary hippocampal cultures on day 7 of neuradapt post-treatment: 1—Intact; 2—Hypoxia; 3—Hypoxia and neuroadapt 1 µM, immediate treatment; 4—Hypoxia and neuradapt 15 µM, immediate treatment; 5—Hypoxia and neuradapt 1 µM, post-treatment; 6—Hypoxia and neuradapt 15 µM, post-treatment treatment. (**a**) The relative number of cells exhibiting Ca^2+^ activity; (**b**) Duration of Ca^2+^ oscillations; (**c**) Frequency of Ca^2+^ oscillations. * versus “Intact”, # versus “Hypoxia”, *p* < 0.05, the Mann–Whitney test.

A major goal in our study is the assessment of functional activity of neuron-glial network in the post-hypoxic period (Figure 5). Registration of calcium dynamics in nervous cells is considered as the most informative approach to study metabolic activity in the elements of neuron-glial networks [44]. Calcium ions are key players in signal transduction, and recording dynamics of Ca^2+^ concentration changes in the cytoplasm allows a precise analysis of neuronal activity and network activity mapping at the cellular and subcellular resolution to be achieved.

The analysis of key characteristics of calcium activity in primary hippocampal cultures reveals that ca. 50.00% (42.22–64.21) of the cells in the intact cultures exhibit Ca^2+^ activity on DIV21, which corresponds to the 7th day of the post-hypoxic period (Figure 5a). The percentage of active cells in the “Hypoxia” group is significantly lower averaging at 25.25% (20.94–31.91). Neuradapt immediate application at 1 μM and 15 μM concentrations maintains the spontaneous calcium activity in nervous cells at the level of intact cultures averaging at ca. 50.00% (43.05–57.58) and 48.39% (34.38–63.16), respectively. A significant neuroprotective effect of neuradapt in the post-treatment scenario is observed for 15 μM concentration: the number of cells exhibited Ca^2+^ activity in this experimental group is 45.71% (36.92–66.65) (Figure 5a).

In the remote period, the modeled hypoxia leads to a significant increase in the duration of Ca^2+^ oscillations compared to the intact group (“intact” 8.82 osc/min (7.36–10.72), vs “hypoxia” 11.85 osc/min (10.24–18.21) (Figure 5b)). An increase in duration of Ca^2+^ oscillations with a decrease in the percentage of cells exhibiting a spontaneous Ca^2+^ activity may indicate the prevalence of astrocytic activity due to the loss in the background activity of neural network. Both the immediate and 2 h post-hypoxia application of 15 μM neuradapt support the normal duration of Ca^2+^ oscillations within the experimental error. 

Thus, neuradapt used in the concentrations studied supports the spontaneous calcium activity at the level of intact cultures in the immediate in vitro hypoxia model. In the post-treatment scenario, only the high concentration of neuradapt (15 μM) shows a pronounced neuroprotective effect.

**Figure 6:**
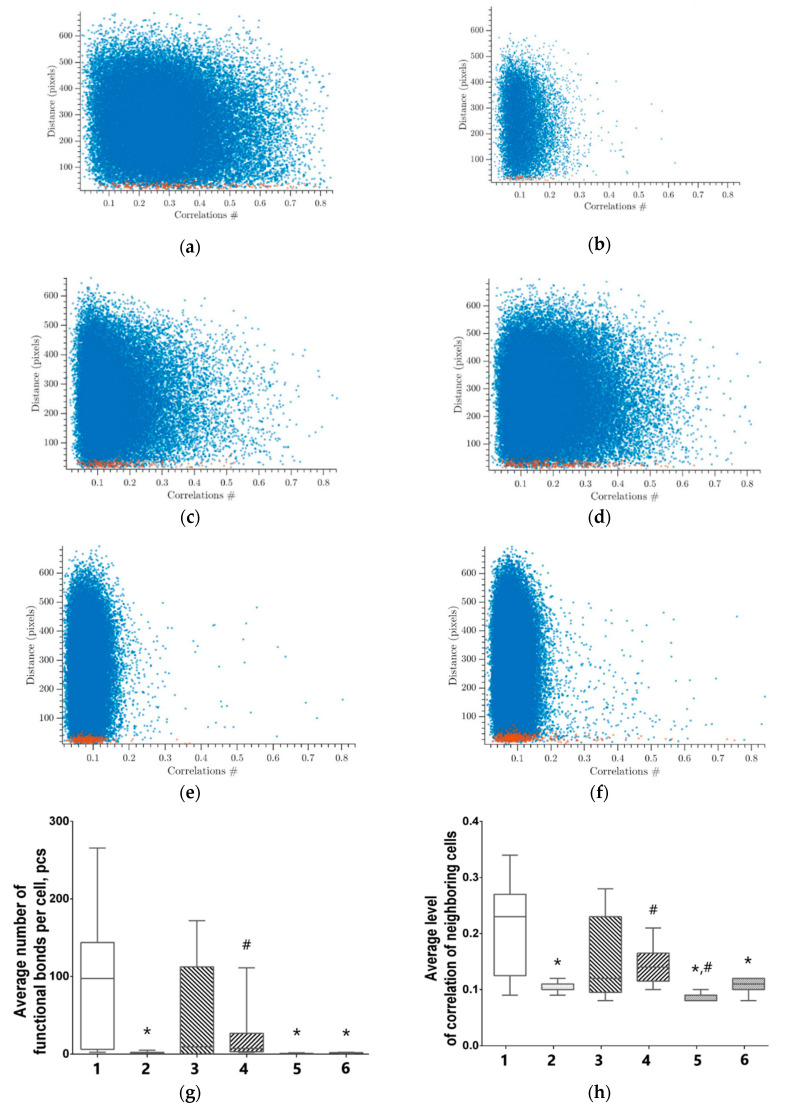

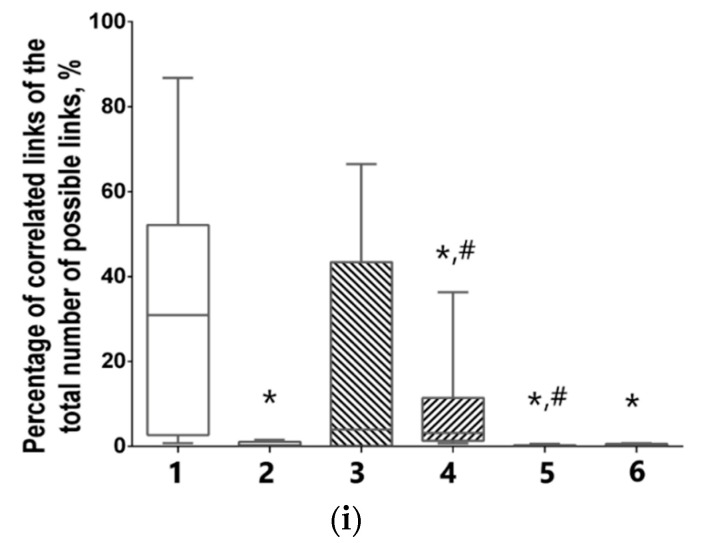
Neuron-glial network activity reorganization in primary hippocampal cultures: 1—Intact; 2—Hypoxia; 3—Hypoxia and neuradapt 1 µM, immediate treatment; 4—Hypoxia and neuradapt 15 µM, immediate treatment; 5—Hypoxia and neuradapt 1 µM, post-treatment; 6—Hypoxia and neuroadapt 15 µM, post-treatment treatment. (**a**–**f**)**,** correlation dependence between spontaneous Ca^2+^ oscillations and cell distance: (**a**) Intact; (**b**) Hypoxia; (**c**) Hypoxia and neuradapt 1 μM, immediate treatment; (**d**) Hypoxia and neuradapt 15 μM, immediate treatment; (**e**) Hypoxia and neuradapt 1 μM, post-treatment; (**f**) Hypoxia and neuradapt 15 μM, post-treatment; (**g**) Average number of functional connections per cell; (**h**) Mean correlation level of adjacent cells; (**i**) percentage of correlated connections from the total number of possible connections. * versus “Intact”, # versus “Hypoxia”, *p* < 0.05, the Mann–Whitney test.

Parameters of Ca^2+^ activity characterizing the connectivity of elements in the neuron-glial network in the post-hypoxic period are of particular interest (Figure 6). The use of network analysis methods provides an opportunity to describe the features of network activity reorganization.

Figure 6a–f presents the dependence between the correlation level and distance of cells pairs in a culture. The adjacent cell pairs with their soma in direct contact, are colored in red. A representative example of intact primary hippocampal culture is shown in Figure 6a. A significant number of cell pairs located far apart from each other have a significant correlation of the signal (0.21 ± 0.08). This indicates an advanced dynamic interaction between distant cells and complex network interactions.

The correlation and dynamic properties of the system are reduced in the post-hypoxic period. The cloud of dots is "shrinking" and the number of significant correlations is decreased both in adjacent and distant cell pairs (Figure 6b). This demonstrates the lowered neural–glial network connectivity and loss of functional connectivity.

**Figure 7:**
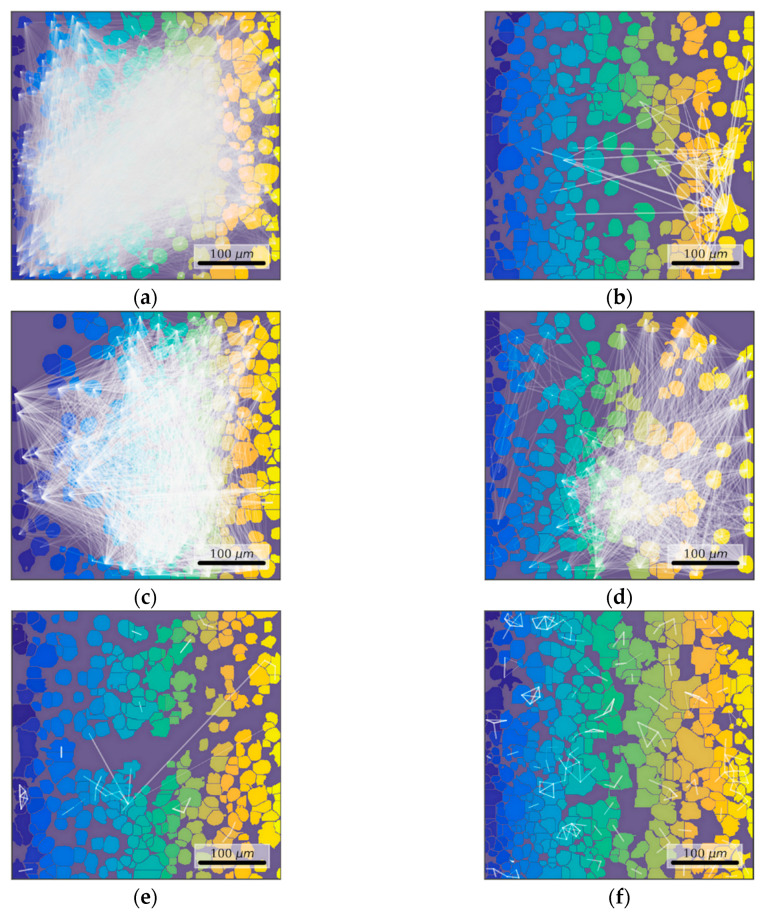
Representative correlation network graphs with a threshold of >0.3. (**a**) Intact; (**b**) Hypoxia; (**c**) Hypoxia and neuradapt 1 μM, immediate treatment; (**d**) Hypoxia and neuradapt 15 μM, immediate treatment; (**e**) Hypoxia and neuradapt 1 μM, post-treatment; (**f**) Hypoxia and neuradapt 15 μM, post-treatment.

Moreover, hypoxia leads to a significant decrease in all studied network characteristics of calcium activity in the primary hippocampal cultures evident of severely disrupted interactions between the network elements (Figure 6 and Figure 7). The average number of functional connections per cell in the “hypoxia” group equals to 0.89 (0.2–2.14) versus “intact” of 97.92 (6.33–143.33). The mean correlation level of adjacent cells is also significantly decreased (“intact” 0.23 (0.15–0.27) vs “hypoxia” 0.10 (0.10–0.10)). The percentage of correlated connections is decreased more than 100 times from the total number of possible connections (“intact” 30.93 (3.54–48.92)% vs “hypoxia” 0.27 (0.09–0.81)%). Therefore, despite the fact that individual elements in the network continue to generate Ca^2+^ oscillations, there is almost a complete absence of coordinated network activity in the primary hippocampal cultures post-hypoxia.

The data on cell connectivity in the culture with the immediate application of neuradapt during hypoxia are of particular interest. The use of neuradapt in hypoxia results in partial preservation of all characteristics of network connectivity. In particular, the immediate application of neuradapt at concentrations of 1 μM and 15 μM during hypoxia significantly increases the number of functional connections per cell relative to the “hypoxia” group: a 35-fold increase for 1 μM (9.54 (0.46–61.51)) and 31.7-fold increase for 15 μM (7.30 (3.27–16.58)), respectively (Figure 6g and Figure 7).

Moreover, the use of the PHD inhibitor supports the maintenance of signal correlation levels in adjacent cells. In the “hypoxia+ 15 μM neuradapt” immediate treatment group, the correlation level is 0.14 (0.12–0.14) being significantly higher than the value for the “hypoxia” group. The number of cells correlated connections relative to the total number of possible connections in this experimental group equals to 3.18% (1.55–3.94).

On the other hand, the network characteristics in the cultures with PHD inhibitor continued treatment post-hypoxia show no difference from the corresponding parameters of the “Hypoxia” group (Figure 6g–i).

Thus, the inhibition of HIF prolyl hydroxylase with neuradapt supports viability and functional activity of nervous cells in in vitro hypoxia models. The use of neuradapt immediately at hypoxia ensures the sustainability of key network characteristics of the functional calcium activity in primary hippocampal cultures. Application of the PHD inhibitor in the post-hypoxic period preserves the viability of a large number of elements in neuron-glial networks despite the loss of the network functional activity. Therefore, the use of neuradapt enables the effective use of activators of synaptogenesis and accelerated reparative processes.

## 4. Discussion

Oxygen deprivation is the major cause for neuronal damage in ischemic diseases. Hypoxia can trigger numerous pathological cascades leading to the loss of nervous cell functions and cell death [45,46]. It was found that preventive PHD inhibition contributes to effective activation of the HIF pathway, which rescues tissues from the damage caused by a subsequent ischemic attack [10,47,48].

The effects of HIF activation on the ischemia-damaged brain are variable and depend on the stroke severity and duration as well as on the amplitude and time-course of HIF activation. Ischemia is far more damaging than hypoxia since in addition to low oxygen it means low glucose. Execution of a HIF-induced survival program requires energy supply, which becomes limited or almost unavailable in oxygen–glucose deprivation models. Immediate treatment with a PHD inhibitor right after ischemia is neuroprotective despite the exact mechanism is controversial and possibly HIF-independent [49]. Pharmacological inhibition of PHD 1 h post-stroke is also reasonably protective, but not as good as in the preventive mode [50]. 

There is no consensus on the role played by HIF activation in neuroprotection induced by PHD inhibitors in the post-hypoxic period [11,51]. In hypoxic state, HIF accumulation may indirectly inhibit the formation of reactive oxygen species through the activity of subsequent transcription targets [52,53]. HIF induces transcription of several anti-apoptotic proteins and stimulates angiopoiesis and erythropoiesis, leading to neuroprotection in ischemic stroke [54,55,56]. At the same time, HIF is also involved in initiation of post-stroke inflammatory reactions, expression of proapoptotic proteins including p53 [57], and blood–brain barrier permeabilization [58]. 

Currently, all available drugs do not specifically inhibit individual PHD isoforms, and supposedly target all three HIF PHD enzymes, despite being developed as PHD2 inhibitors. It is assumed that PHD2 is the most important PHD isoform regulating HIF levels [59]. However, several studies reveal that PHD2 inhibition leads to a decrease in neurite growth and deterioration of neurological functions recovery after ischemic brain injury [60]. PHD1 inhibition has been directly implicated into neuronal survival in oxidative stress [61]. HIF PHDs catalyze hydroxylation of more than a dozen client proteins beyond HIFs. Hence, PHD inhibitors will affect numerous pathways in addition to those induced by HIF stabilization. A plausible candidate substrate for post-insult protection is a PHD3 substrate, transcription factor ATF4, as discussed in [12]. However, the exact molecular mechanism responsible for the cell survival induced by HIF PHD inhibitors post-hypoxia awaits its resolution.

The ability of PHD inhibitors to maintain cell viability in the post-hypoxic period can be considered as the first step in a multicomponent therapy aimed at minimizing the ischemic injury, and can be followed by methods and/or drugs activating de novo synaptogenesis. Therefore, there is a need to conduct additional studies on the post-treatment effects of HIF prolyl hydroxylase inhibition on nervous system adaptation to hypoxic injury. Our study reveals that a PHD pan-inhibitor, neuradapt, within the 0.5–20 μM concentration rage is non-toxic for in vitro primary hippocampal cells cultures. Moreover, pharmacological PHD inhibition by neuradapt, a low-molecular weight compound, being used in normobaric hypoxia, not only prevents neuronal cell death, but in addition, supports cell viability and the main characteristics of calcium activity. Our data are in agreement with the results obtained on rat primary brain cortex cells cultures and murine hippocampal HT-22 cells [61,62].

The application of network analysis of functional calcium activity with cellular resolution shows that an episode of acute hypoxia disrupts network interactions between the nerve cells up to the almost complete network negation and thus, supports the data obtained previously with the use of multielectrode arrays [24,63]. The demonstrated effect of network activity disruption cannot be explained by cell death, as the cell viability in the culture remains at a sufficiently high level (72–77%). However, the maintenance of neuronal viability is not sufficient to preserve the neuronal functions. It is well known that each neuron forms thousands of synaptic contacts with the other cells [64]. Oxygen deprivation leads to some synaptic contacts being reduced or silenced [65,66], which results in the loss of functionally significant elements in neuron-glial networks, and, as a consequence, in the impairment of their functional activity, including memory and cognition [67,68].

## 5. Conclusions

Our study clearly demonstrates that, in order to develop effective methods for neuroprotection, it is not sufficient to monitor the cell viability, but also necessary to assess both the functional state of nerve cells and the level of network interactions between elements of neuronal networks. The work presents important results revealing that the use of a PHD inhibitor, neuradapt, immediately at the hypoxia modeling step preserves the cell connectivity in primary hippocampal cultures at a significantly higher level than in the untreated “hypoxia” group. These results make the studied compound extremely promising for development of medications boosting brain cell adaptation to hypoxic injury. When the novel PHD inhibitor is used in a post-treatment scenario, the parameters of spontaneous calcium activity such as the number of active cells and the frequency of Ca^2+^ oscillations have been preserved, but the functional interconnections between the cells have been disturbed and the network connectivity lost. Nevertheless, one may assume that these changes are reversible and associated with a temporary decrease in synaptic activity [69]. The described benefits of neuradapt, a specific inhibitor of PHDs, make it an attractive candidate for the first line of medications in a combinatorial approach aimed at increasing the adaptive capacity of brain neural networks. That is an exciting area for upcoming research.

## Figures and Tables

**Table 1:** Neuradapt toxicity for primary hippocampal cultures assayed on day 7 post-treatment.

Group	Number of Viable Cells, %
Intact	91.25 ± 0.75
Control (DMSO)	89.71 ± 1.26
Neuradapt 0.5 μM	93.02 ± 1.65
Neuradapt 1 μM	93.40 ± 1.31
Neuradapt 2 μM	92.49 ± 1.54
Neuradapt 5 μM	90.01 ± 1.55
Neuradapt 10 μM	90.86 ± 1.80
Neuradapt 15 μM	92.15 ± 1.78
Neuradapt 20 μM	89.95 ± 1.23
Neuradapt 30 μM	81.06 ± 2.37 *

* versus “Intact”, *p* < 0.05, one-way ANOVA and Tukey post hoc test.

**Table 2:** Cell viability of primary hippocampal cultures on day 7 post-hypoxia modeling with immediate neuradapt application.

Group	Number of Viable Cells, %
Intact	91.24 ± 0.75
Control (DMSO)	89.71 ± 1.26
Hypoxia	76.61 ± 2.67 *
Hypoxia + DMSO	81.29 ± 3.82 *
Hypoxia + neuradapt 0.5 μM	91.21 ± 1.97 ^#^
Hypoxia + neuradapt 1 μM	89.55 ± 1.75 ^#^
Hypoxia + neuradapt 5 μM	90.43 ± 1.73 ^#^
Hypoxia + neuradapt 10 μM	89.37 ± 2.37 ^#^
Hypoxia + neuradapt 15 μM	92.67 ± 0.49 ^#^
Hypoxia + neuradapt 20 μM	92.78 ± 1.13 ^#^

* versus “Intact”, # versus “Hypoxia”, *p* < 0.05, two-way ANOVA and Tukey post hoc test.

**Table 3:** Cell viability of primary hippocampal cultures on day 7 with daily neuradapt application started 2 h post-hypoxia.

Group	Number of Viable Cells, %
Intact	91.06 ± 0.97
Hypoxia	73.11 ± 1.76 *
Hypoxia + Neuradapt 1 μM	80.85 ± 1.41 *^,#^
Hypoxia + Neuradapt 15 μM	80.70 ± 1.19 *^,#^

* versus “Intact”, # versus “Hypoxia”, *p* < 0.05, two-way ANOVA and Tukey post hoc test.

## Supplementary Materials

The following are available online at https://www.mdpi.com/2076-3921/9/8/662/s1, Table S1: Primers for real-time PCR.
